# Psychological impact of COVID-19 pandemic on Parkinson's disease patients

**DOI:** 10.1016/j.heliyon.2022.e09604

**Published:** 2022-06-03

**Authors:** Muhammad Tufail, Changxin Wu

**Affiliations:** Institute of Biomedical Sciences, Shanxi University, Taiyuan, 030006, China

**Keywords:** COVID-19, Parkinson's disease, Emotional stress, Physiological stress, Cognitive stress

## Abstract

COVID-19 is a highly infectious disease caused by SARS-CoV-2. It causes respiratory tract infection that ranges from mild to lethal. The present study aimed to investigate the psychological impact of COVID-19 on Parkinson's disease (PD) patients. A questionnaire about the emotional, physiological, and cognitive stress symptoms was designed in the present study. A total of 94 cases and 188 controls participants filled out the questionnaire. The participants include 70.2% male and 29.8% female in both cases and controls. 27.6% of the participants were aged 18–40 years old, 33.0% were aged 41–60 years old, and 39.4% were above 61 years old. In the present study, we found that the emotional symptoms of stress were common in PD patients. Fear about own and family health was significantly higher in PD patients. A significant number of PD patients were feeling depressed; the major reason was the COVID-19 and being a PD patient, While job difficulties and COVID-19 pandemic was the main reason for feeling depressed in the control group. Constant worrying due to COVID-19 was also more common in PD patients than in the control group. Among the physiological symptoms of stress, low energy, Restlessness, clenched jaw and avoiding others were significantly higher in PD patients. Among the cognitive symptoms of stress, racing thoughts, forgetfulness, and more nervous behaviours were common in PD patients. This study concludes that PD patients face a psychological burden due to the COVID-19 pandemic, which needs proper attention.

**Psychological impact**

The current study presents the psychological impact of the COVID-19 pandemic on PD patients. This study revealed that PD patients face more psychological burdens than the general population, suggesting that PD patients are at high risk of developing psychological problems which need proper attention.

## 1 Introduction

During past outbreaks of infectious diseases, the ongoing COVID-19 (Coronavirus Disease 2019) has manifested profound and wide-ranging psychosocial impacts on individuals, communities, and nations [1]. In previous outbreaks, there was a significant psychological impact on uninfected community members and poor psychosocial and coping responses to the outbreak of infectious diseases and persistent fear of contracting the disease [2]. In the current state of knowledge, there is a scarcity of information on the psychological impact of the pandemic, especially in terms of mental health effects of the public, patients with neurodegenerative disease. Several studies have been conducted on the COVID-19 outbreak, focusing on identifying its epidemiology, clinical features, genomic characterization, clinical features, transmission mode and its route, reservoirs, and incubation period, Monitoring symptomatic and clinical outcomes, including mortality and survival rates; preventing virus spread; and managing global health governance [3, 4, 5, 6, 7]. As a result of COVID-19, a comprehensive research project on psychological health and mental well-being must be undertaken.

Due to the mental health problems caused by the current pandemic infection COVID-19, which has medical as well as psychosocial repercussions, it has triggered excessive fear, discrimination and other mental health issues around the world [8], including anger, stress, anxiety, and fear are common depressive symptoms [9], that increase the complications and aggravations of chronic diseases [10].

The COVID-19 pandemic has already significantly impacted global health and social-economic security. There is a greater death rate among the elderly, particularly those with underlying conditions. In addition, COVID-19 causes anxiety and fear for various reasons, resulting in prolonged coping mechanisms [11]. A high level of mental health problems is associated with the enforced social isolation resulting from the COVID-19 lockdown. Research suggests that catastrophizing, loneliness, and perceived mood changes are negative factors, while positive refocusing and contamination fear are positive factors. SEM analysis revealed that intolerance of uncertainty and loneliness adversely impacted mental health. More specifically, maladaptive emotion regulation strategies mediated the relationship between intolerance of uncertainty, contamination fear, and loneliness. Whereas adaptive emotion regulation strategy mediates the relationship between social support and mental health through contamination, fear, and loneliness [12]. Numerous neurodegenerative diseases, characterized by the loss of neurons and/or myelin sheaths, impact hundreds of millions of senior people worldwide [13]. These illnesses are generally severe and necessitate long-term care. COVID-19 pandemic is most likely to affect the elderly population. COVID-19 has been studied in individuals with several neurodegenerative disorders, including Alzheimer's, Parkinson's, prion disease, and amyotrophic lateral sclerosis. In addition, the possible mechanisms of SARS-CoV-2 infection in the aetiology of neurological disorders were also described [14].

Parkinson's disease (PD) is the second most prevalent neurological condition afflicting millions of people worldwide [15]. The most common PD symptoms are tremors, stooped posture, and slow movement. These symptoms may not be present in the early stages, although their prevalence increases as the disease advances [16]. Parkinson's disease (PD) can be inherited [17] or sporadic [16]; however, nowadays, sporadic instances account for roughly 90% of cases. Spontaneous Parkinson's disease (PD) risk, on the other hand, is well understood to be influenced by a combination of genetic and environmental risk factors. Some researchers have looked into the connection between PD and the environment [18]. Although we know much about how to control the disease, it is still affecting normal life, such as it has financial and psychological effects. The present study aimed to investigate the psychological impact of the COVID-19 pandemic on Parkinson's disease in the Pakistani population.

## 2 Methodology

### 2.1 Study population

Pakistan is a developing country [19]. This population-based study was conducted in Khyber Pakhtunkhwa, Pakistan, formally known as North-West Frontier Province (NWFP). It is the smallest province of Pakistan geographically but is the third-largest province economically and population-wise. The life expectancy rate in Pakistan is 66.95 years [20].

### 2.2 Study design

Before initiation of the study, ethical committee approval was obtained from Shanxi University, and consent forms were obtained from the participants. This study comprised residents of KPK who were aged 18 years or older. This study covered both newly diagnosed and previously diagnosed PD patients. A case was classified as a PD patient diagnosed by neurologists according to the UK Parkinson's Disease Society Brain Bank guidelines. The cases and control groups were statistically matched, and two controls were enrolled for each case Table 1. A sample of controls of the same regions in KPK province was chosen in the study; individuals from other provinces were excluded. Control groups were not associated with one another or with PD patients. The definition of controls was individuals of the same regions that reproduced the cases. Individuals with a history of stress or depression or stress symptomology before the outbreak of COVID-19 were excluded. In the final selection, 94 PD patients and188 controls were enrolled.

**Table 1:** Demographic characteristics of cases and controls.

Variables	Cases (%)	Controls (%)
Gender		
Male	66.0 (70.2)	132 (70.2)
Female	28.0 (29.8)	56 (29.8)
Age		
18–40	26 (27.6)	50 (27.6)
41–60	31 (33.0)	64 (33.0)
61 and above	37 (39.4)	74 (39.4)

### 2.3 Questionnaire design and data collection

The questionnaire has been divided into three major parts. The first part was about emotional stress, the second part was about physiological stress, and the third part covered cognitive stress. The questions about emotional stress were “fearing about own and family health, feeling depressed and main reason of being depressed, difficulty in relaxing mind, frequent nightmares and constant worrying”. The questions about the physiological stress were “low energy, frequent headache, aches and pain, frequent cold, restlessness, upset stomach, rapid heartbeat, nervousness, dry mouth, clenched jaws and avoiding others”. Cognitive stresses were covered by the following questions “racing thoughts, forgetfulness, unable to focus on things, poor judgment, being pessimistic, appetite, eating pattern and sleep changes, avoiding responsibilities and insomnia”.

378 participants were contacted via phone between September 2021 to December 2021. Two controls were enrolled for each case. A total of 235 control participants were tried to contact. 17 participants did not answer the call, 19 were busy in some activities and did not provide time to answer the questions, 07 were unwilling to participate, and 04 were excluded. The case group consisted of Parkinson's disease (PD) patients. The participant's mobile contact information was already available. A total of 127 PD patients were tried to contact. Among 127 patients, 09 did not answer the phone call, 13 were busy at work and could not answer the questionnaire, while 11 refused to participate, and 16 patients were demised (According to their family).

### 2.4 Statistical analysis

Data were coded in SPSS version 25. The statistical analysis was divided into two categories: the descriptive analysis was used to study the demography of participants, while Chi-square (X^2^) was used to study the association of psychological impact of COVID-19 pandemic on PD and control group. Statistical significance was determined by a *p*-value ≤ 0.05. The demographic characteristics of cases and controls were represented in percentage. In order to determine whether the difference between observed data and expected data was due to chance or if it could be explained by the variables studied, the Chi-square test was used.

## 3 Results

This study included 94 cases and 188 controls. In the present study, 70.2% of participants were male, and 29.8% were female. Moreover, 39.4% of the participants were aged 61 and above years old, followed by 41–60 years old (33.0%) and 18–40 years old (27.6%) (Table 1).

The questionnaires were filled out from the patients and controls to determine the impact of stress symptomology on PD patients due to COVID-19. The questionnaire consists of three main parts. The first part of the questionnaire was about emotional stress. Anger, fear, worry and sorrow are all normal responses to stress. They are all classified as emotional stress and are a normal response to the pressures of everyday life. Pandemic quarantine indeed involves many social, emotional, psychological, and physical modifications, which may increase an individual's distress. A quarantined or lonely environment can result in psychiatric and physical injury, social isolation, and loneliness [21, 22]. We found that some of the emotional stress impact PD patients significantly. We found that 70.2% of PD patients and 51.6% of the control group were worried about their own and family health (*p* = .003). Among PD patients, 46.8% of patients felt depressed, while 13.3% of controls felt depressed (*p* < .0001). The major reason for being depressed in cases was COVID-pandemic and being a PD patient (*p* < .0001), while difficulties of job due to COIVD-19 pandemic was the main reason for depression in the control group (*p* = .024). Difficulty in relaxing the mind (*p* < .0001) and constant worrying (*p* = .001) was another emotional impact of the COVID-19 pandemic on PD patients. We did not find a significant impact of being feeling anger and frequent nightmares on PD patients (Table 2).

**Table 2:** Prevalence of emotional stress symptomology in cases and controls.

Variables	Cases (%)	Controls (%)	OR (95% Cl)	p-value (X^2^)
Fear of own and family health	66 (70.2)	97 (51.6)	2.21 (1.30–3.74)	**.003**
Feel angry	05 (5.3%)	11 (5.9)	0.90 (0.30–2.68)	.856
Feeling depressed	44 (46.8%)	25 (13.3)	5.73 (3.19–10.29)	**.0001**
Reason of feeling depressed				
COVID-19	02 (2.1)	02 (1.2)	2.02 (0.28–54.58)	.476
COVID-19 and job difficulties	03 (3.2)	21 (11.2)	0.26 (0.07–0.90)	.024
COVID-19 and PD	37 (39.4)	03 (1.6)	40.0 (11.89–134.7)	**.0001**
Difficulty in relaxing mind	37 (39.4)	24 (12.8)	4.43 (2.44–8.04)	**.0001**
Frequent nightmares	02 (2.1)	06 (3.2)	0.65 (0.13–3.33)	.723
Constant worrying	26 (27.7)	21 (11.2)	3.04 (1.60–5.76)	**.001**

The second questionnaire was about the physiological stress impact of the COVID-19 pandemic on PD patients. Environmental stress, intrinsic developmental stress, and aging are three forms of physiological stress that can challenge an individual's cells or organisms [23]. A global health disaster such as COVID-19 causes psychological stress and anxiety over time in people from every society globally. This pandemic has caused great stress that will live long in people's minds, including Parkinson's disease patients [24]. This study revealed that a significant number of PD patients faced low energy than the control group (*p* = .002). Aches and pains were also more common in PD patients (16.0%) than in the control group (5.9%). Moreover, 24.5% of PD patients faced restlessness while only 3.2% of controls suffered (*p* < .0001). Clenched jaw (*p* = .026) and avoiding others (*p* < .0001) were also common in PD patients. This study found no significant difference of frequent headaches, frequent cold, upset stomach, rapid heartbeat, nervousness, and dry mouth between the cases and control group (Table 3).

**Table 3:** Prevalence of physiological stress symptomology on cases and controls.

Variables	Cases (%)	Controls (%)	OR (95% Cl)	p-value (X^2^)
Low energy	17 (18.1)	12 (6.4)	3.23 (1.47–7.10)	**.002**
Frequent headache	02 (2.1)	05 (2.7)	0.79 (0.15–4.18)	.787
Aches and pain	15 (16.0)	11 (5.9)	3.05 (1.34–6.95)	**.006**
Frequent cold	02 (2.1)	01 (0.5)	4.06 (0.36–45.41)	.218
Restlessness	23 (24.5)	06 (3.2)	9.82 (3.84–25.14)	**.0001**
Upset stomach	01 (1.1)	10 (5.3)	0.19 (0.02–1.51)	.082
Rapid heartbeat	03 (3.2)	01 (0.5)	6.16 (0.63–60.09)	.075
Nervousness	09 (9.6)	08 (4.3)	2.38 (0.88–6.39)	.077
Dry mouth	02 (2.1)	06 (3.2)	0.65 (0.13–3.33)	.612
Clenched jaw	04 (4.3)	01 (0.5)	8.31 (0.91–75.43)	.026
Avoiding others	64 (68.1)	74 (39.4)	3.28 (1.94–5.54)	**.0001**

In the third part of the questionnaire, we asked questions about the cognitive impact of the COVID-19 pandemic on PD patients. Cognitive functions, also known as executive functions, are cognitive processes that operate through anticipating and establishing goals, creating plans and programs, initiating activities and mental operations, and monitoring task performance. Executive functions, such as organizing thoughts, are interrupted by excessive stress if the prefrontal cortex is affected [25]. Both healthy adults and patients with neurodegenerative diseases have experienced worsening motor and cognitive functions because of distancing and confinement at home during the COVID-19 outbreak. The lockdown has negatively affected the physical and mental health, the quality of life, daily activities, and diet behaviour due to the decrease in physical activity, the stoppage of the recovery intervention and the social distance it imposed [26, 27]. We found that 33.0% of patients had racing thoughts, while 9.0% of the controls reported racing thoughts (*p* < .0001). Forgetfulness was also common in cases (35.1%) than the control group (3.7%) (*p* < .0001). PD patients were more being pessimistic (24.5%) than the control group (10.1%) (*p* = .001). Change in sleep was reported in 16.0% of cases, while 4.8% of controls reported change in sleep (*p* = .002). This study found no significant impact of insomnia, changes in sleeping and eating patterns, inability to focus on things and poor judgment, avoiding responsibilities, and more nervous behaviour (Table 4).

**Table 4:** Prevalence of cognitive stress symptomology in cases and controls.

Variables	Cases (%)	Controls (%)	OR (95% Cl)	p-value (X^2^)
Racing thoughts	31 (33.0)	17 (9.0)	4.95 (2.56–9.56)	**.0001**
Forgetfulness	33 (35.1)	07 (3.7)	13.98 (5.88–33.24)	**.0001**
Unable to focus on things	12 (12.8)	17 (9.0)	1.47 (0.67–3.22)	.332
Poor judgment	10 (10.6)	18 (9.6)	1.12 (0.49–54)	.778
Being pessimistic	23 (24.5)	19 (10.1)	2.88 (1.47–5.61)	**.001**
Change in appetite	06 (6.4)	11 (5.9)	1.09 (0.39–3.06)	.860
Avoiding responsibilities	11 (11.7)	10 (5.3)	2.35 (0.96–5.77)	.054
More nervous behavior	12 (12.8)	14 (7.4)	1.81 (0.80–4.10)	.146
Change in sleep	15 (16.0)	09 (4.8)	3.77 (1.58–8.99)	**.002**
Change in eating pattern	04 (4.3)	09 (4.8)	0.88 (0.26–2.94)	.841
Insomnia	04 (4.3)	17 (9.0)	0.44 (1.46–1.36)	.149

## 4 Discussion

Coronavirus disease 2019 (COVID-19) is caused by the 2019 novel coronavirus. In COVID-19; CO stands for corona; VI stands for the virus, and D stands for disease [28]. As a result of the ongoing COVID-19 pandemic, people with Parkinson's disease (PD) may experience many consequences, including social distancing measures and lifestyle changes, increasing psychological stress and worsening symptoms. Researchers have recently uncovered a link between external stressors (the COVID-19 pandemic) and worsened Parkinson's symptoms by inciting psychological distress and influencing lifestyle modifications (reduced physical activity) [29, 30]. The COVID-19 outbreak may be stressful for people around the world. Fear due to COVID-19 may be overwhelming and can cause emotional, physiological, and cognitive stress. In the present study, we aimed to investigate the psychological impact of the COVID-19 pandemic on PD patients. In the current study, 94 PD patients and 188 controls participated. Several factors are associated with developing stress, such as financial problems, loss of a job, or chronic illness. A recent study from Khyber Pakhtunkhwa, Pakistan, reported a significant association between Parkinson's disease and depression [31]. Another study also reported that healthcare workers are at high risk of developing stress, depression, and anxiety due to the outbreak of SARS [32]. The outbreak of COVID-19 may increase the risk of stress directly, such as being afraid of their health or their loved one's health, or it may increase the risk of stress indirectly as several people are losing their job and many of them have financial issues.

The current study found that the COVID-19 pandemic has a psychological impact on PD patients. Despite the fact that loneliness negatively affects the quality of life, to our knowledge, this may be the first time that this association has been demonstrated in a PD population in PD patients from Pakistan. This study found that emotional stress, including fear about own health and family health, difficulty in relaxing mind, constant worrying, and feeling depressed, have a significant impact on PD patients. Individuals with loneliness rated all symptoms as more severe than those with no loneliness. The greatest differences between the two cohorts were found in withdrawal/loss of interest, motivation/initiative, depression, and anxiety [33, 34]. There have been reports that up to 22.8% of patients with PD have suffered worsenings in their clinical condition during the outbreak of COVID-19. The epidemic profoundly changes individuals' routines [35, 36]. In recent years, a growing body of research has suggested that many people with Parkinson's might experience cognitive and motor inflexibility due to the depletion of nigrostriatal dopamine, an essential component of the pathophysiology [37, 38]. Two explanations are possible for this phenomenon. One is that dopamine-dependent adaptation is thought to function as a flexible mechanism to cope with environmental stress [37, 39]. Additionally, increased psychological stress can temporarily worsen motor symptoms, such as tremors, freezing of gait, or dyskinesias, and may decrease the effect of dopaminergic medications, which need proper attention [34, 40, 41, 42, 43].

Moreover, job difficulties due to the COVID-19 pandemic were significantly associated with depression in the control group. A previous study has reported that financial problems are associated with developing stress [44]. Israel's COVID-19 study demonstrates various psychological characteristics and perceived dangers. People may feel safer when they distance themselves from others, but this can also lead to increased emotions of loneliness, tension, and irritation, leading to troubles in a variety of areas of one's life [45].

On the 11th of March, a new coronavirus disease (COVID-19) became widespread. Additionally, COVID-19 resulted in a wide range of physiological stress. There is a pressing public need to find a solution to the problem of helping people recover from the effects of post-traumatic stress disorder [46]. Our study revealed that PD patients are at high risk of Physiological stress due to the COVID-19 pandemic compared to the general population. The most common physiological stress variables were having low energy, aches and pain, restlessness, clenched jaws, and avoiding others.

The third major part of our questionnaire was about cognitive symptomology. Some of the cognitive stress symptoms were significantly common in PD patients, such as racing thoughts, forgetfulness, being pessimistic, and change in sleeping. A recent study also reported that the COVID-19 pandemic has the possibility of altered cognitive function [47].

Moreover, this study sheds light on how the COVID-19 pandemic may negatively impact PD patients in the KPK population. It is, therefore, essential to understand which of these factors leads to an increase in the psychological burden on PD patients in this population and to develop an appropriate prevention strategy. However, our study has some limitations, including small sample size, although this sample size is adequate for the study population. Parkinson's disease is rare, and therefore it was almost impossible to study large sample size. Another reason for the small sample size was that we had recruited only participants who did not have depression symptomology before the pandemic of COVID-19. In future research, practice, and policy development, the impact of social isolation on poor outcomes in PD needs further research, particularly considering the ongoing social distancing regulations associated with COVID-19 pandemic.

## 5 Conclusion

This study concluded that the COVID-19 increased the burden of psychological stress on PD patients. The COVID-19 pandemic increases the burden of emotional, physiological, and cognitive stress. The psychological impact of COVID-19 on PD patients needs proper attention and health priorities for policymakers, authorities, and academicians who should adopt important strategies to reduce the burden of the COVID-19 pandemic on PD patients. Further study is warranted in other regions of Pakistan.

## Declarations

### Author contribution statement

Muhammad Tufail: Conceived and designed the experiments; Analyzed and interpreted the data; Wrote the paper.

Changxin Wu: Conceived and designed the experiments; Wrote the paper.

### Funding statement

This research did not receive any specific grant from funding agencies in the public, commercial, or not-for-profit sector.

### Data availability statement

Data included in article/supp. material/referenced in article.

### Declaration of interest's statement

The authors declare no conflict of interest.

### Additional information

No additional information is available for this paper.

## Appendix A Supplementary data

quest quest
