# Supplementary material for: Psychological impact of COVID-19 pandemic on Parkinson's disease patients
Source: Heliyon. 2022 Jun 3;8(6):e09604. doi: 10.1016/j.heliyon.2022.e09604 (PMC9166998; doi:10.1016/j.heliyon.2022.e09604)
Supplement: quest [file mmc1.pdf]

Gender	Male/Female
Age	
Are you Fear of own and family health during COVID-19 pandemic?	Yes/No
Do you Feel angry during COVID-19 pandemic?	Yes/No
Do you Feeling depressed during COVID-19 pandemic?	Yes/No
What is the Reason of feeling depressed?	COVID-19<br>COVID-19<br>job difficulties<br>COVID-19 and PD
Do you face difficulty in relaxing mind during COVID-19 pandemic?	Yes/No
Do you have frequent nightmares during COVID-19 pandemic?	Yes/No
Are you constant worrying during COVID-19 pandemic?	Yes/No
Do you have low energy during COVID-19 pandemic?	Yes/No
Do you have frequent headache during COVID-19 pandemic?	Yes/No
Do you have aches and pain during COVID-19 pandemic?	Yes/No
Do you have frequent cold during COVID-19 pandemic?	Yes/No
Do you have Restlessness during COVID-19 pandemic?	Yes/No
Do you have Upset stomach during COVID-19 pandemic?	Yes/No
Do you have Rapid heartbeat during COVID-19 pandemic?	Yes/No
Are you feel Nervousness during COVID-19 pandemic?	Yes/No
Do you have Dry mouth during COVID-19 pandemic?	Yes/No
Do you have Clenched jaw during COVID-19 pandemic?	Yes/No
Are you Avoiding others during COVID-19 pandemic?	Yes/No
Do you have Racing thoughts during COVID-19 pandemic?	Yes/No
Are you facing Forgetfulness during COVID-19 pandemic?	Yes/No
Are you Unable to focus on things during COVID-19 pandemic?	Yes/No
Do you have Poor judgment during COVID-19 pandemic?	Yes/No
Are you Being pessimistic during COVID-19 pandemic?	Yes/No
Are you facing Change in appetite during COVID-19 pandemic?	Yes/No
Are you Avoiding responsibilities during COVID-19 pandemic?	Yes/No
Do you have More nervous behaviour during COVID-19 pandemic?	Yes/No
Is there a Change in sleep during COVID-19 pandemic?	Yes/No
Is there a Change in eating pattern during COVID-19 pandemic?	Yes/No
Do you face Insomnia during COVID-19 pandemic?	Yes/No
